# The Role of Mealtimes in Fostering Language Development and Aligning Home and School Learning: Protocol for a Multi-Method Study of Preschool Children in Rural Kenya and Zambia

**DOI:** 10.2196/36925

**Published:** 2022-07-05

**Authors:** Henriette Zeidler, Claire Farrow, Megan Jarman, Grace Koteng, Barnabas Simatende, Danielle Matthews, Haatembo Mooya, Laura R Shapiro, Pamela Wadende

**Affiliations:** 1 School of Psychology College of Health and Life Sciences Aston University Birmingham United Kingdom; 2 Department of Educational Psychology School of Education and Human Resource Development Kisii University Kericho Kenya; 3 Department of Psychology Humanities and Social Sciences University of Zambia Lusaka Zambia; 4 Department of Psychology University of Sheffield Sheffield United Kingdom

**Keywords:** language development, nutrition, preschool education, school, education, home, environment, academic, children, student, language learning, language, caregiving, responsive caregiving, speech, child-directed speech, nutritional level

## Abstract

**Background:**

The association between school and home is fundamental to sustainable education: parents’ understanding of the school’s priorities and teachers’ understanding of their pupils’ home environment are both vital for children to remain in school and succeed academically. The relationship between parents and teachers is closest in preschool settings, providing a valuable opportunity to build bridges between home and school. In this protocol paper, we outline our planned methods for identifying beneficial home and school behaviors.

**Objective:**

Our project aims to identify culture-specific structures and behaviors in home and school settings, which influence the quantity and quality of child-directed speech and identify positive experiences that can help improve children’s linguistic development and nutrition.

**Methods:**

Using a mixed methods approach and focusing on early language learning, nutrition, and responsive caregiving, we will video-record and analyze mealtime language and eating behaviors at home and in school, targeting 80 preschool children and their families in rural Kenya and Zambia. In addition, we will assess children’s language skills through audio recordings and use questionnaire-based interviews to collect extensive sociodemographic and dietary data.

**Results:**

Between the start of our project in January 2020 and the end of December 2021, we had collected complete sets of sociodemographic, observational, and food recall data for 40 children in Kenya and 16 children in Zambia. By the end of May 2022, we had started data collection for an additional 24 children in Zambia and transcribed and coded approximately 85% of the data. By the end of September, 2022, we plan to complete data collection, transcription, and coding for the entire sample of 80 children across both countries. From September 2022 onwards, we will focus on analyzing our language data, and we hope to have results ready for publication in early 2023. By relating children’s language outcomes and nutritional intake to the observed mealtime behaviors, we hope to identify practices that increase the quantity and quality of child-directed speech and improve children’s nutritional intake.

**Conclusions:**

Good nutrition and the promotion of language learning are key issues in early childhood development. By using a cross-cultural approach, combining a variety of methods, and working closely with stakeholders and policy makers throughout the project, we hope to find and share best practices for improving children’s linguistic outcomes and nutrition and lay the foundation for the development of practitioner networks and parent outreach programs.

**International Registered Report Identifier (IRRID):**

DERR1-10.2196/36925

## Introduction

Sustainable education builds on a strong connection between school and home settings. In order for children to remain in school and succeed academically, it is important to have not only teachers who understand their home environment but also parents who are familiar with their children’s educational settings [1]. However, home and school remain largely separated in many African countries [2]. At home, children start out by learning about all aspects of life in an integrated way, which is quite different from the typical school setting with a strict separation of disciplines [3,4]. As a result, children are likely to find it difficult to adapt to the school environment and the required focus on different areas. Indeed, many African children do not meet expected school standards for their age; in Kenya 6-to-13–year-old primary school children are roughly 2 years behind the expected academic levels for numeracy and literacy skills by international standards [5], and this gap is even more pronounced in rural areas of low socioeconomic status [6]. Thus, there is significant potential to raise educational standards by better aligning school with home as part of focused early-year interventions across rural sub-Saharan Africa.

Two key drivers of children’s educational success that are determined both by home and school are good nutrition [7,8] and a responsive caregiving environment that supports rich linguistic interactions [9-11]. Mealtimes provide an ideal context to evaluate both of these factors as one can detail not only regular dietary intake but also exposure to potentially sophisticated language for a meaningful duration of time each day [12,13]. Studies to date suggest that children’s diets and the structure of home mealtimes vary considerably both between and within African countries [14]. In this study, we propose to measure this variation in families based in Kenya and Zambia and relate this to later cognitive outcomes in order to identify potential bridges between home and school settings, which could support their development.

Regarding dietary variation, we will make use of the mealtime settings to explore children’s nutritional intakes and identify best practices for providing equally healthy and nutritious diets for all children at home. While children’s nutritional status has improved throughout the last decade, food insecurity and malnutrition still affect millions in Kenya and Zambia. In 2014, it was reported that 26% of Kenyan children were stunted, 11% were underweight, and 4% were wasted. In Zambia numbers were even higher, with 40% of children stunted, 15% underweight, and 6% wasted [15].

Stunting is strongly related to parental education and income and is more common in children of mothers who have not completed primary school or are in the lowest wealth quintile [16,17]. In addition, children in rural Kenya often have deficiencies in iron, calcium, and zinc [18], all of which are thought to influence children’s cognitive development [19].

Concerning linguistic variation, substantial differences can arise from the context in which families share meals. While children in rural areas often eat alone or with their mothers, parents who have spent more time in school might prefer family meals where everyone eats together. As a result, children’s communicative experiences vary in many ways. Families in higher-socioeconomic status settings are more likely to have homes with electricity and a TV set in the sitting room, which might interfere with mealtime interactions and lead to delayed language development [20]. Although these factors suggest a counterintuitive pattern where children in low–socioeconomic status families may have greater exposure to family communication during mealtimes, the benefits gained largely depend on the quality of these communicative interactions. Previous work has shown that the semantic and pragmatic content of caregiver-child interactions varies enormously between social settings [21] and cultures [22]. An important aspect of our project is thus to identify culture-specific structures and behaviors that influence the quantity and quality of child-directed speech in potentially language-rich settings.

Our research addresses 3 major challenges for early development in low-income communities: improving children’s nutrition, improving children’s linguistic development, and aligning school education with children’s everyday experience [23,24].

**Figure 1:**
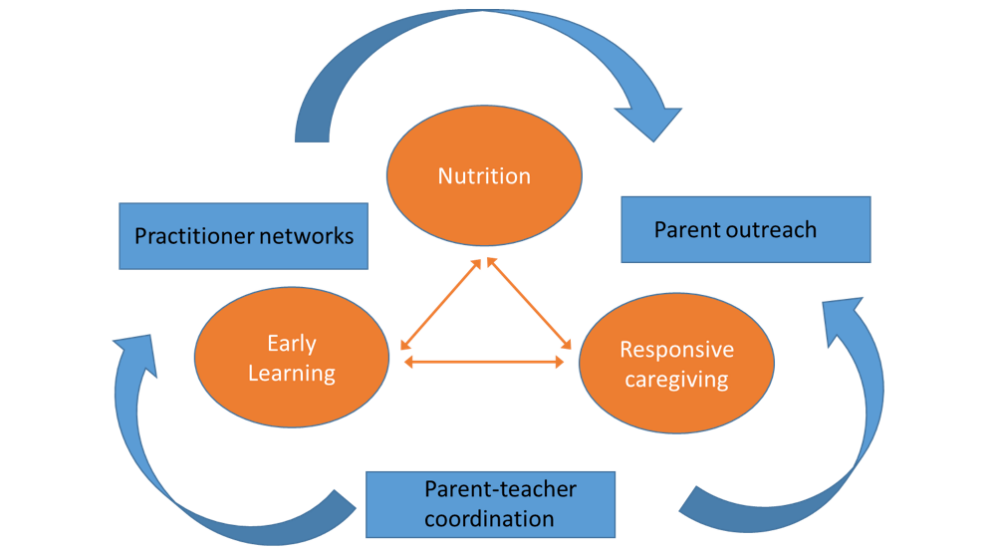
Priorities for targeting key domains of early childhood education and care and aligning preschool and home settings. Developed from Black et al [25].

By observing children’s and caregivers’ behavior and language use in home and school settings, we aim to identify positive experiences that teachers can build on in the classroom and that parents can use at home. Ultimately, we hope to initiate a collaborative network that involves teachers from Early Child Development and Education (ECDE) centers along with families to improve 3 domains of nurturing care (nutrition, early learning, and responsive caregiving) for children in their charge. Figure 1 illustrates the overarching goals of our project [25].

The specific goals of this protocol provide the first steps toward our overarching aim. Specifically, our aims were as follows:

1. To observe child language exposure and child language outcomes across a 2-year time span: (1) measure the quantity and quality of child-directed and child-accessible speech within home and school mealtime language and identify mealtime practices associated with individual differences in language exposure; and (2) examine whether individual differences in language exposure are related to developments in the target child’s language proficiency;
2. To observe mealtime behaviors at home and collect dietary data to identify practices that support healthy child nutrition; and
3. To identify best practices that can cross-inform home and school settings about supporting early cognitive development to foster educational success.

## Methods

### Ethics Approval

The project has been reviewed by the ethics review unit at Baraton University of Eastern Africa (B22972019), by the University of Zambia ethics committee (2020-Nov-111) as well as by the Aston University research ethics committee (#1547), and we have obtained permission to conduct this research from the relevant authorities in Kenya and Zambia.

### Setting and Participant Selection

Our participants are 4-to-7–year-old children, their caregivers, siblings, peers, and others who live and eat with our target children.

In Kenya, we are recruiting 40 children from 2 rural primary schools in Laikipia County, and in Zambia, we are recruiting the same number of individuals from a total of 4 rural primary schools, amounting to a total of 80 children across the 2 countries. Our Kenyan participants are predominantly Kikuyu, and our Zambian participants are from Tonga and Lozi communities.

From each participating school, we are also recruiting one preschool teacher to become part of our research team. Throughout the project, our teacher-researchers will help establish contacts with local families and assist with data collection.

### Data Collection

#### Mealtime Observations

We will observe each child for a total of 4 mealtimes within a period of 3 months: twice at home and twice in school. In addition, we will have one pre–data collection observation per family and 2 per school to familiarize all participants with the recording situation. We know that a high level of meal-to-meal variability across different eating episodes is likely [26], and we will attempt to minimize this by observing lunchtime meals for all participants and having multiple observations for each child. In all cases, the research team will attempt to record “typical” mealtime interactions (defined by parents and teachers), using digital recording equipment placed covertly from the child’s view. For each observation, we will ask adult participants whether the mealtime was unusual or normal, rating it on a scale from 1 (very different from usual) to 5 (completely normal).

#### Child Language Outcomes

In order to test for associations between mealtime language and behaviors with language outcomes, we will measure children’s linguistic skills 1 year after our initial baseline observations. We will provide children with a wordless picture book, and following a short period of familiarization, we will ask them to recount the story to an assistant over the phone. In addition, we will ask children to provide a free narrative of recent events or experiences. In both cases, we will measure the quantity of word types and tokens produced by the child.

#### Dietary Recall

In addition to our observations, we will use a 24-hour dietary recall to assess children’s food and beverage intake in more detail. Caregivers will be asked to recall a list of all food and drinks consumed in the last 24 hours—quantity, preparation methods, and meal times—for a total of 3 days (2 weekdays and 1 Sunday). Photographs of the foods and drinks described in the recalls will be collected by the caregiver using a smartphone to assist with validation and portion size estimation. The dietary recall procedure will follow the multi-pass method that has been adapted to local standards in line with recommendations from the GloboDiet-Africa team [27] and extensively piloted with local preschool teachers and research assistants.

#### Background Information

In order to put our findings into perspective, we will measure covariates that tend to be associated with child language and educational attainment [5], including household income, parental education, child gender, and child age [5,28]. To that end, we have developed a comprehensive sociodemographic questionnaire, which will be administered to our participating families on the first day of our visit. In order to minimize the burden on our participants and ensure that they do not have to be literate, local research assistants will read the questions in local languages and record the answers. We will also measure the number of adults and siblings living in participating family homes as this is likely to influence language exposure.

In addition, we will collect data about children’s reading opportunities and practices at home. To complement our dietary data, we will also measure children’s weight and height. Child height and weight will be collected in schools by a team of teachers or research assistants trained in using measuring materials. All measures will be taken by one team member and confirmed by a second member of the team. Children will be weighed and measured in their school uniforms with shoes removed.

### Data Management

The study will be conducted in accordance with data sharing agreements established prior to data collection, which are aligned with each university’s policies, standard operating procedures, and regulatory requirements for data protection, storage and security, and secure data sharing across sites at Aston University, Kisii University, and the University of Zambia. Each participant will be assigned a unique registration number. A master list linking each participant’s registration number to identifying details will be stored separately from the deidentified data. Data will be securely stored on paper (inside a locked filing cabinet) and electronically (in password-protected folders) at each site. In addition, electronic data will be stored on a secure server at Aston University.

### Protocol Adaptation Owing to the COVID-19 Pandemic

Prior to the COVID-19 outbreak, we had successfully recruited 16 families in Kenya and had begun collecting initial home and school mealtime observations. As soon as COVID-19 restrictions were introduced at each site (March 2020), we suspended all the observations we had planned and stopped all in-person contact between the research team and community members in order to avoid potential transmission of the virus. Using already established links between participating families and research assistants, we were, however, able to continue collecting food recall data over the phone. Once schools were reopened, we continued our recruitment activities with eligible families through local ECDE teachers.

Additionally, as a result of the pandemic, we also decided to collect language outcome data using phone story book retell activities with children in order to minimize direct contact. We worked with teachers and local assistants from our participating communities to select an appropriate story book that the children would find relatable, which was also likely to elicit rich language. In order to further minimize contact, we distributed the story books via participating ECDE teachers during normal everyday interactions with participating families in the community. To ensure that none of the children had previous experience with the book, we handed them out in sealed envelopes and asked parents to open them only at the time of the recording. Teachers familiarized parents with the planned procedures when distributing books and also used this opportunity to hand out masks and sanitizers and sensitize participants about health-related behaviors during the pandemic.

### Data Coding Plan

#### Linguistic Measures Taken From Mealtime Language Observation Data

Participants are likely to be multilingual and speak in their native languages (Kikuyu and Tonga or Lozi at the Kenyan and Zambian sites, respectively) as well as use official languages (Kiswahili or English in Kenya and English in Zambia). Since the use of native languages is encouraged in the preschool setting, we expect to find a similar mix of languages in both settings. We will transcribe 20 minutes of conversation from each video recording, starting from when the target child has received his or her meal. All transcriptions will be performed by local research assistants who are fluent in the expected languages, using ELAN software [29]. In addition, the transcribers will provide an English translation of the conversation in order to enable the entire research team to understand the content. Coding and analyses will, however, be based on the original languages used and will be carried out by research assistants who are fluent in the local languages. We will code for utterance direction and accessibility, as well as communicative function of utterances. In addition, we will measure (1) word types and tokens produced by the child, (2) adult word types and tokens within overheard speech segments, (3) adult word types and tokens within child-directed segments [30], and (4) word types and tokens spoken by other children, within segments directed at our target child (see Multimedia Appendix 1 for the language coding scheme).

#### Behavioral Measures Taken From Mealtime Observation Data

In order to code behavioral variables from our recordings, we have developed a culturally appropriate coding scheme for family mealtimes, using an adapted version of a coding scheme developed by Mutoro et al [31] for children living in low-income areas in Nairobi. Mutoro’s original scheme was developed for younger children; hence, our coding scheme was adapted to suit the behaviors and interactions of older children. The new scheme codes for the mealtime setup and seating arrangements (including who is eating with the child and whether bowls are shared), the location of the child (eg, sitting on the floor or at the table), and the presence of any distractions (such as the radio, TV, or animals). The scheme codes for behaviors include caregivers’ encouragement to eat, prompting to eat, negativity or use of force, as well as child behaviors such as interest in food, interactions with other children, mood, distractibility, and any challenging behaviors during the meal. The scheme also codes for the overall tone of the mealtime and considers how much the child eats from among the foods offered. The scheme was codeveloped by academic experts (including native experts) in eating behavior, parenting, child development, and observational methodologies, alongside local teachers and local research assistants who were able to advise on culturally specific meanings of specific behaviors, which were used to adapt the scheme and to appropriately reflect the nature of the interactions (see Multimedia Appendix 2 for the full behavioral coding scheme).

#### Nutritional Assessment

From the recall data for each child, we will calculate the amount of food in grams per day and then use the Food Composition Tables from Kenya [32] to calculate mean daily nutrient intakes. These will then be compared to nutrient intake recommendations as outlined in the joint Food and Agriculture Organization of the United Nations (FAO) and World Health Organization (WHO) 2002 report [33] to calculate nutrient adequacy ratios. The joint FAO/WHO 2004 report on energy requirements [34] will be used to determine adequate or inadequate energy (in kcal) intakes based on age, sex, and weight. Child height and weight will be converted to weight-for-height for age Z scores (WHZs) and height-for-age (HAZ) Z scores, using the WHO AnthroPlus software version 1.0.4 [35]. Children will be classified as being underweight if their WHZs are ≤−2 SD. Having overweight or obesity will be defined as WHZ>2 SD and >3 SD, respectively. Stunting will be defined as HAZ≤− 2 SD.

### Data Analysis Plan

#### Language and Behavioral Analysis

For language and behavioral analysis, we shall carry out the following:

- Compare scores from the mealtime coding scheme to identify whether there are significant differences between home and school settings for each of the potential predictors (eg, caregiver sensitivity and distractors) and examine the nature of mealtime structure (ie, number of people and seating arrangement) to highlight key differences;
- Examine variability in mealtime language measures within and across school or home settings and, if appropriate, merge data within a setting. We will then examine which of our predictors and covariates are significantly associated with our mealtime child language measures and our child-language outcomes;
- Conduct a series of hierarchical regressions (controlling for significant covariates) to examine which behavioral factors are predictive of each of our mealtime language and child-language outcome variables.

#### Dietary Analysis

For the dietary analysis, we will carry out the following:

- Compute average daily nutrient intake estimates and explore adequacy ratios of key vitamins and minerals. These include energy, calcium, iron, magnesium, zinc, selenium, vitamin A (retinol equivalents), thiamin, riboflavin, niacin, folate, vitamin B12, and vitamin C. In addition, we will calculate dietary diversity and food variety scores and assess levels of stunting and wasting;
- Assess whether the rich observational data validates parentally reported dietary intake for the children. As for most children, a 24-hour recall was completed the day following home mealtime observations we can compare foods consumed during the mealtime with those reported in the 24-hour recall;
- Explore whether children’s diets and mealtime experience are related to social and background variables (eg, number of people and seating arrangement), as well as behavioral variables that have previously been associated with food intake (eg, caregiver responsiveness, prompting, and use of distractions);
- Compare home and school observations to see how interactions differ depending on the context and explore what behaviors support better nutrition and language development for children;
- Explore the role of siblings and other children who are more involved in mealtime interactions in rural Africa compared to Western countries, and assess whether the quality and quantity of interactions with other children is beneficial for child nutritional adequacy and language outcomes.

## Results

Our study started in February 2020, which coincided with the onset of the COVID-19 pandemic in Kenya. Despite the resulting challenges, we managed to continue our work owing to our close relationships with the communities and by adapting our aforementioned methods. As of May 2022, we had collected complete sets of sociodemographic, observational, and food recall data for all 40 children in Kenya, and we will have transcribed and coded the data by the end of August 2022. In addition, we have collected and transcribed language outcome data for 14 Kenyan children and intend to collect and transcribe those data for the remaining 26 children by September 2022. The nutritional data for a subset of our Kenyan participants, which had been collected during the onset of the COVID-19 pandemic, has already resulted in a published paper [36]. Our results show that reduced access to marketplaces, financial restrictions, and limited availability of products led to changes in the types and quantities of food that parents were able to provide during the pandemic.

In Zambia, we had collected and transcribed sociodemographic and observational data for 16 children by the end of December 2021 and added food recall and language outcome data from them by the end of May 2022. In addition, we recruited another 24 children in March-April 2022 and hope to complete data collection for the entire sample by the end of August 2022.

From September 2022 onward, we will focus on analyzing our language data, and we hope to have results ready for publication in early 2023.

## Discussion

### Principal Findings

Key to the success of early childhood development programs is good nutrition and the promotion of language learning through responsive caregiving [37]. This project is novel in its focus on both language development and nutrition. These two aspects of child development have tended to be studied separately in previous research, thus neglecting the potential for each area to shape the other. Our research findings will advance theories about how early developmental experiences during mealtimes influence language development and nutrition. Some factors we identify will likely be universal across cultures (eg, the importance of quality and quantity of child-directed speech on children’s language outcomes) and other factors will likely vary across cultures (eg, the effect of men and women eating together on child-directed speech). The extent to which these factors are common across Kenya and Zambia will indicate the generalizability of our findings to other rural communities in Africa and worldwide. Outputs will include the following: (1) identification of culturally relevant caregiver behaviors (including those of siblings and peers) that enhance the quantity and quality of child-directed speech during mealtimes and, in turn, promote child language; (2) assessment of the influence of caregiving practices and mealtime structures on children’s nutrition; and (3) identification of best practices at home and in school, which can cross-inform the 2 settings.

### Comparison With Prior Work

Nutrition in the early years of life is crucial to children’s health, growth, and cognitive development. Previous research with children in sub-Saharan Africa suggests that diets are mainly cereal-based and have low levels of dietary diversity [38]. In many cases, children also have inadequate energy intakes and do not consume enough fat or micronutrients such as calcium, zinc, riboflavin, or vitamins A and B_12_ [38,39]. Identifying best practices for raising children’s nutritional levels in low-income, rural settings, which can easily be adapted for use across similar communities, can have a lasting positive impact on children’s lives.

A focus on young children’s exposure to and use of language during mealtimes is also valuable for 2 reasons: (1) mealtimes provide a rich source of social interaction, and (2) the focus on eating is likely to be perceived as a natural setting for communication. We know that from early childhood, the quantity and quality of child-directed speech is important for language learning, and that naturalistic measures of type and token frequency are a valuable way of measuring linguistic experience and proficiency across different cultures and languages [40], provided all speakers who engage with the child are counted. There is strong empirical evidence that siblings play a more important role in language socialization in rural African families than in Western societies [41,42]. Serpell [43] notes that preadolescent children are often left in charge of their younger siblings in multi-age play groups; hence, including older siblings in our observations will greatly increase the scope of existing work.

### Strengths and Limitations

While we have attempted our best to collect naturalistic data, video observations can always create slightly artificial situations. We have attempted to alleviate this problem by adding dummy recordings and carefully preparing participants in prerecording interviews, but it is still possible that we have not captured the most natural behaviors. In addition, we decided to focus on lunch times in order to not interfere too much with our participants’ private lives; nonetheless, suppers might have provided a more typical setting for family mealtimes. Lastly, the number of both participants and observations was restricted by time and available funding for our project. Extending both would certainly lead to a richer and more powerful data set.

### Future Directions

Our project is thus only a small step toward understanding how children’s social environment shapes their developmental outcomes. A longitudinal setup following children’s development from their first year of life throughout preschool would provide much deeper and more comprehensive insights. In addition, adding participants from different socioeconomic, cultural, and linguistic backgrounds would allow us to draw much wider conclusions. In terms of interventions, it would be interesting to focus on peer-to-peer interactions and identify situations and ways in which children in mixed age groups can support each other’s learning, thus further bridging the home and school environments.

### Dissemination Plan

We hope to publish at least 3 additional papers toward the end of our project in early 2023: one focusing on children’s linguistic outcomes, one targeting children’s nutritional status, and one qualitative paper describing and comparing children’s home and school environments across sites.

In addition, we will share our results with colleagues and stakeholders through meetings, conferences, and social media. Using a bottom-up approach, we will discuss our results with parents and teachers from our local communities and share their feedback with policy makers at local, national, and international levels. To that end, we have built a close network of collaborators including school representatives, educationalists, language and community experts, and relevant government officials in Kenya and Zambia.

## Appendix

Multimedia Appendix 1 Language coding scheme.

Multimedia Appendix 2 Behavioral coding scheme.

## Abbreviations

ECDE: Early Childhood Development and Education
FAO: Food and Agriculture Organization of the United Nations
HAZ: height-for-age Z score
WHO: World Health Organization
WHZ: weight-for-height for age Z score

## Data Availability

The data that support the findings of this study will be openly available in the UK Data Archive [44].
