# Supplementary material for: The Role of Mealtimes in Fostering Language Development and Aligning Home and School Learning: Protocol for a Multi-Method Study of Preschool Children in Rural Kenya and Zambia
Source: JMIR Res Protoc. 2022 Jul 5;11(7):e36925. doi: 10.2196/36925 (PMC9297130; doi:10.2196/36925)
Supplement: Multimedia Appendix 1 [file resprot_v11i7e36925_app1.docx]

**Language Coding Scheme**

**Coding time**

- Start coding immediately after food has been handed to target child – this should be the same time the transcription starts.
- To code the first few utterances, you may need to watch some of the footage prior to the coding start point to provide context.
- Do not code during any periods of offshot (i.e., where there is an annotation in the offshot tier indicating the target child left the room).
- Code for 20 minutes from the starting point (which is when transcription should also end).

**Coding tiers**

Underneath each speaker tier (except the target child tier) there are 2 tiers for coding:

1. **utterance direction & accessibility**
2. **communicative function**
3. **Utterance direction & accessibility**

***TIER: xxx_direction***

Each speaker tier (except the target child) has a linked tier beneath it (a dependent tier), labelled *_direction* with a prefix identifying the speaker. For example, *Mot_direction* is linked to the Mother tier where all the mother’s utterances are to be coded for utterance direction and accessibility. This involves categorising each utterance based on whom the utterance is directed to/intended for (i.e., whether the utterance is directed to the target child/group including target child or another person/other people), and whether the utterance is accessible/relevant to the target child or inaccessible (i.e., difficult to understand or follow).

To select a category, click on the transcribed utterance in the desired speaker tier to highlight in purple. Then right click in the respective *xxx_direction* tier below the speaker utterance you are coding, and click on ‘New Annotation Here’ or double click in the space. The categories will appear in a drop-down list. Select the appropriate category for the utterance you are coding.

***Coding categories***

Code all utterances made by all speakers – excluding the target child – in the respective *xxx_direction* tier by categorising the utterances into 6 mutually exclusive, exhaustive categories:

- **TC_accessible**

Select this category if:

- - the utterance was directed to target child either specifically or to a group that includes the target child;
  - AND the target child is likely to hear and understand/follow/make sense of the utterance.
- **TC_inaccessible**

Select this category if:

- - the utterance was directed to target child either specifically or to a group that includes the target child;
  - AND the target child would likely not be able to hear and/or understand/follow/make sense of what is being said. For example, a topic of conversation where the content of what was said is at a level above what the target child would be able to understand (i.e., difficult for a young child to understand). This will likely be rare in speech directed to the target child.
- **Other_accessible**

Select this category if:

- - the utterance was directed to another person, group of people that does not include the target child, an animal, another person on the phone, to themselves (e.g., thinking out loud or reading something such as a leaflet aloud);
  - AND the target child would likely be able to overhear and understand/follow/make sense of the utterance.

If an utterance is not directed to another person/people present (e.g., directed to an animal, someone on the phone, to themselves), make a note in the notes tier describing the intended recipient of the utterance.

- **Other_inaccessible**

Select this category if:

- - the utterance was directed to another person, group of people that does not include the target child, an animal, someone on the phone, to themselves (e.g., thinking out loud or reading something such as a leaflet aloud);
  - AND the target child would likely not be able to hear and/or understand/follow/make sense of what is being said. For example, a topic of conversation where the content of what was said is at a level above what the target child would be able to understand (i.e., difficult for a young child to understand). For example, parents talking about problems at work.

If an utterance is not directed to another person/people present (e.g., directed to an animal, someone on the phone, to themselves), make a note in the notes tier describing the intended recipient of the utterance.

- **Unclear**

Select this category when it is difficult to determine utterance direction and accessibility.

- **Inaudible**

Select this category when an entire utterance is inaudible or when too much of the utterance is inaudible to determine utterance direction and accessibility (i.e., who the utterance is intended for and whether it is accessible). This would be likely for utterances transcribed with a number of ‘xxx’ codes.

***Notes on determining utterance direction***

- To help determine if an utterance is directed to the target child/a group of people that includes the target child, or to another person/people, look out for cues such as gaze direction (who the person is looking at), context, utterance content, gestures, body language, proximity to the target child etc.
- Sometimes it is clear that an utterance is directed to another person, but the target child responds (which is a typical part of conversation). If this happens, still code as *Other_accessible/Other_inaccessible* (depending on accessibility of the conversation)*.*

***Notes on determining accessibility***

- An utterance is likely accessible if it is:
  - within reasonable proximity to the target child that they would have had the opportunity to listen to/overhear the utterance;
  - AND the target child would likely be able to understand/follow/make sense what is being (i.e., the topic of conversation was at a level the target child would be able to understand).
- If someone is speaking in English (more than the usual for the home) and the child would be unlikely to understand, this would be coded as *Other_inaccessible.*
- Another example of speech that would likely be hard for the child to understand is a parent reading a medical procedure leaflet aloud in English. This would be coded as *Other_inaccessible.*
- Other types of utterances likely to be *Other_inaccessible* are:
  - a person talking to someone else in another room and it is too quiet to hear from the audio recorder (so likely similar for the target child).
  - when the target child and a sibling are having a conversation, and mother (for example) and another sibling are having a conversation – it is unlikely the target child will be able overhear the conversation between mother and second sibling.

**2. Communicative function**

***TIER: xxx_function***

Each speaker tier (except the target child) has a linked tier beneath it (a dependent tier), labelled *_function* with a prefix identifying the speaker. For example, *Bro1_function* is linked to the Brother_1 tier. Here you will **code ONLY the utterances that were categorised as *TC_accessible/TC_inaccessible*** (i.e., utterances directed to the target child) and categorise each utterance based on the function it served (e.g., a response to the target child, behaviour management etc.).

**Code only utterances directed to the target child** (i.e., all utterances coded as *TC_accessible/TC_inaccessible*) in the respective *xxx_function* tier by categorising the utterances into the following 8 mutually exclusive, exhaustive categories:

**A) RESPONSE** statements will only include **reactions to the target child's utterance**

- **A1) Response_eliciting =** utterances that are:
  - **in response** to the target child’s previous utterance and therefore semantically contingent and/or logically related to the target child’s previous utterance;
  - **AND topic continuing** i.e., the utterance refers to something that was referred to in the target child’s previous utterance)
  - **AND conversation-eliciting** i.e., utterances intended to elicit a verbal response, which include questions, clarification requests/requests for repetition, requests for information, statements that elicit talk e.g., “tell me what happened”, prompts e.g., “tell me more”, and prompts for a reply to a previous question – the desired response is a verbal response from the target child.
- **A2) Response_expansion** = utterances that are:
  - **in response** to the target child’s previous utterance and therefore semantically contingent and/or logically related to the target child’s previous utterance;
  - **AND topic continuing** i.e., the utterance refers to something that was referred to in the target child’s previous utterance)
  - **AND expands** on the target child’s previous utterance.

Utterances that expand on the target child’s previous utterance are utterances that either:

- - 1. expands on what the target child was saying or doing, providing additional information about an object, action or topic in an effort to continue the conversation, or encourages the child to think about what they said in their utterance that was responded to. In other words, utterances in response to the target child’s utterance that directly builds on the child’s topic to provide more information or explanation – it maintains the conversation on the topic.
    2. repeats the child’s utterance but alters what the child said to make it grammatically correct i.e., adding additional words to make a more grammatically correct phrase from what the child produced.
    3. phrases what the child said in grammatically different way. So rather than producing the child’s phrase in the same grammatical form e.g., the child’s utterance is a statement and the parent’s utterance is also a statement), the parent’s utterance phrases the child’s in a different grammatical form such as a question or negation. For example, the child says “Cat sleep” and the parent says, “Is the cat sleeping?” or “The cat isn’t sleeping”.

**NB. utterances that fall under descriptions ii. and iii. are more common with very young children so will be less likely to occur.**

**If a response is conversation-eliciting as well as an expansion of what the child said, code as conversation-eliciting and make a note in the notes tier.**

- **A3) Response_other** = utterances that are:
  - **in response** to the target child’s previous utterance and therefore semantically contingent and/or logically related to the target child’s previous utterance;
  - **AND EITHER topic continuing** i.e., the utterance refers to something that was referred to in the target child’s previous utterance, or the utterance was a paraphrase of the target child’s previous utterance, or was an answer to the child’s question **BUT is not a conversation-eliciting response or an expansion response**
  - **OR NOT topic continuing** i.e., responses that are essentially “topic-less”. For example, acknowledgements of child utterances such as interjections and expressions of agreement e.g., ‘oh’, ‘okay’, ‘right’ ‘mmmh’, ‘umhmm’, ‘I see’, ‘yes’ but not in answer to a child’s question as this would be topic continuing.
  - This category can also include responses to a target child’s utterance that are intended as behaviour management, prohibitions, punitive/disciplinary statements (and likely simultaneous behaviour). These can be topic continuing. For example, the parent says, “what were you told by the teacher to do after you wash your hands?” “After you wash your hands what do you do?” and the child responds, “this way” whilst demonstrating how to wash hands, and the parent then responds, “show them how to scrub your hands”. Or not topic continuing e.g., if the child says “how am I going to get out?” whilst stuck behind a table and attempting to get up, and the parent responds by saying, “sit down”.

***Notes on coding responses***

- If a person says a series of consecutive utterances that are all in response to the target child’s utterance, code all of those utterances as a response (under the relevant response category) e.g., Child: “Muriithi told me to go back”. Parent: “Why?” “That’s what Muriithi told you? = code both as *Response_eliciting.*
- Responses to the target child’s utterance can be from different people i.e., a number of people can respond to what the target child said. For example, Child: “My leg is feeling pain”. Father responds: “You have to sit down” and Mother responds: “Is it pressing you?” – both are responses to the target child’s utterance.
- When coding responses, this is a good opportunity to transcribe any easy-to-miss utterances such as exclamations, filled pauses (i.e., sounds often made whilst people pause) etc.

**B) INITIATE** statements which are **directed to the target child only or a group including the target child:**

- **B1) Initiate_eliciting** = utterances that are:
  - intended to elicit a verbal response from the **target child**, which includes questions, requests for information, questions about the child’s ongoing actions, statements that elicit talk e.g., “tell me what happened at school today”, and prompts to answer previous questions – the desired response is a verbal response from the target child;
- **B2) Initiate_statement** = utterances that are:
  - **NOT in response** to the target child’s previous utterance i.e., the utterance is semantically unrelated to the target child’s previous utterance; and can be directed at **anyone in the group**
- **B3a) Initiate_directive** = utterances that are:
  - intended as behaviour management, prohibitions, punitive/disciplinary statements;
- **B3b) Initiate_directive_food** = utterances that are:

- intended as behaviour management, prohibitions, punitive/disciplinary statements; AND related to food - i.e. *Eat up! Finish your food!*

- **B4) Initiate_Other** = utterances that do not fall under one of the above categories (e.g., calling/saying the child’s name to get their attention).

**C) FOOD-RELATED COMMENTS (TARGET CHILD ONLY)**

For all **utterances made by the target child**, please identify the ones which are about food, and specify whether these are positive/ negative/ neutral comments.

**D) UNCLEAR**

Select this category when the utterance is audible, but it is difficult to determine the communicative function

**E) INAUDIBLE**

Select this category when an entire utterance is inaudible or when too much of the utterance is inaudible to determine its function. This would be likely for utterances transcribed with a number of ‘xxx’ codes.
