# Supplementary material for: The Role of Mealtimes in Fostering Language Development and Aligning Home and School Learning: Protocol for a Multi-Method Study of Preschool Children in Rural Kenya and Zambia
Source: JMIR Res Protoc. 2022 Jul 5;11(7):e36925. doi: 10.2196/36925 (PMC9297130; doi:10.2196/36925)
Supplement: Multimedia Appendix 2 [file resprot_v11i7e36925_app2.docx]

**Behavioural coding inventory: mealtime observations**

**Background codes (structure and setting)**

- **Meal-length:** Note time food given to child and time child finishes food or leaves table – calculate mealtime length approximately in minutes and seconds. Start of meal = when child is given food, end of meal is when food is taken away or child leaves food to not return.
- **Location of the primary caregiver and child** during meals (1= sat on floor, 2 = sat on sofa or chair no table, 3 = sat on sofa or chair with a low table, 4= sat at a higher table, 5 = other, specify).

Code for child and caregiver. If participants move around code where they are most of the time.*For School observations, code only child's location*

- **Number of people:**
  - Number of people present/ around during mealtime. Specify for each gender and if adults or older/ younger children. People will come and go, count all those who interact with the people who are eating at any point*. - Home observations only*
  - Number of people who are sitting with child during mealtime. Specify for each gender and if adults or older/ younger children. *- Home observations only*
  - Time teacher(s) present. State for how much of the 20-min coding period a teacher is present (all the time/most of the time/sometimes/rarely/never) - *School observations only*
- **Distractions**
  - Is child engaged with another activity while eating, (e.g. playing a game during meal) If yes specify.
  - Is the television or radio on (yes/ no) - *Home observations only*

#### Behavioural codes

#### These codes should be made based on an average of what you have seen across the period being coded. Code for the main person who is feeding/supervising the child. Where there are 2 people who are co-supervising the child (e.g. mother and older sibling) code for them both.

#### Caregiver(s) list: List who is supervising the target child during the meal (prompting the child to eat, watching over progress of meal time). This could be one person (e.g. mother) or several people (e.g. mother, sister).

#### Caregiver encouragement (defined as smiling at the child, praising the child, chatting to the child or singing with the child).

All the time Most of the

time time Sometimes Rarely Not at all

#### Caregiver prompting child to eat their food (e.g. comments such as eat up, finish your soup, have another bite).

All the time Most of the

time time Sometimes Rarely Not at all

#### Caregiver negativity including threats to the child if they do not eat food, telling the child off, force feeding the child, or silence during the meal. Pay attention to the tone of the caregiver.

All the time Most of the

time time Sometimes Rarely Not at all

#### Caregiver distraction: e.g. if their attention was diverted from the child during the meal.

All the time Most of the

time time Sometimes Rarely Not at all

#### Child-to-child interactions (amount of all interactions going on between children present during mealtime - target child does not have to be included in these)

All the time Most of the

time time Sometimes Rarely Never

#### Involvement of target child in child-to-child interactions (how much is the target child involved in interactions between children)

All the time Most of the

time time Sometimes Rarely Never

#### Mood of children's interactions (average from across the mealtime)

Negative, More negative Neutral Mostly positive, Very happy,

full of arguments than positive good interactions laughter

- - **Child interest in food:** A child is very interested in food if they readily eat independently showing enjoyment of food, neutral if they eat independently but require some prompting by the caregiver and not interested if they are avoiding the food and having to be prompted to eat throughout the meal.

Not at Less Neutral Moderately Very

all interested interested interested interested

- - **Child’s mood** overall

Crying Sad Calm Happy Very happy

- - **Child distraction**. Can be seen if the child’s attention is diverted for example because they are playing with an object, playing with someone else or looking elsewhere

All the time Most of the

Time time Sometimes Rarely Not at all

- - **Food refusal.** Did the child refuse to eat?

All the time Most of the

Time time Sometimes Rarely Not at all

- - **Overall tone of the mealtime**

Negative, More negative Neutral Mostly positive, Very happy,

full of arguments than positive good interactions laughter
